# The Evolution of Hypertension Guidelines Over the Last 20+ Years: A Comprehensive Review

**DOI:** 10.7759/cureus.31437

**Published:** 2022-11-13

**Authors:** Endurance O Evbayekha, Okelue E Okobi, Tobechukwu Okobi, Emeka C Ibeson, Jane N Nwafor, Oyintoun-emi Ozobokeme, Adedoyin Olawoye, Ihuoma A Ngoladi, Maureen G Boms, Faridah A Habib, Babatunde O Oyelade, Caroline C Okoroafor, Vivian N Chukwuma, Kesena B Alex, Evidence E Ohikhuai

**Affiliations:** 1 Internal Medicine, St. Luke's Hospital, Chesterfield, USA; 2 Family Medicine, Arizona State University, Tempe, USA; 3 Family Medicine, Lakeside Medical Center, Belle Glade, USA; 4 Internal Medicine, BronxCare Health System, New York City, USA; 5 Internal Medicine, Maimonides Medical Center, New York City, USA; 6 Internal Medicine, University of DC, Silver Spring, USA; 7 Medicine, Central Michigan University College of Medicine, Mount Pleasant, USA; 8 Family Medicine, Private Practice, Calgary, CAN; 9 Clinical Research, University of Alabama at Birmingham, Birmingham, USA; 10 Family Medicine, Alberta International Medical Graduates Association (AIMGA), Calgary, CAN; 11 Cardiology, Emory University, Atlanta, USA; 12 Family Medicine, University of Calabar, Calabar, NGA; 13 Internal Medicine, University of Illinois at Chicago/Advocate Christ Medical Center, Chicago, USA; 14 Faculty of Medicine, University of London, London, GBR; 15 Public Health, Jackson State University School of Public Health, Jackson, USA

**Keywords:** heart health, cardiovascular disease, hypertensive heart disease, review, guidelines, hypertension

## Abstract

Hypertension is the most common modifiable risk factor for cardiovascular and cerebrovascular diseases. In the last two decades, the guidelines have evolved tremendously from areas with no recommendations for screening or treatment to targeted recommendations for some at-risk groups. We sought to go through the literature that provided guidelines for the management of hypertension at any point in time over the last 22 years from 2000 to 2022. We searched four databases: PubMed, Embase, Google Scholar, and Cochrane, using specified search terms. The keywords used were "hypertension" and "guidelines." We combined them using the Boolean operators (AND, OR) and searched for articles. A total of 2461 publications were initially identified; 348 publications were excluded after screening for full-text availability. The full-text articles were further filtered based on title and abstract screening. Following this, a total of 1443 articles were excluded. The remaining 670 full-text articles were assessed for eligibility. Of the 670 full-text articles, 480 were excluded based on exclusion criteria, and following the full-text article screening, 190 articles met the final inclusion criteria. Most of these guideline evolutions concerned establishing and adjusting thresholds for the subgroups of the elderly population and patients with diabetic kidney disease, chronic kidney disease, and stroke. Furthermore, the medications of choice are now guided by the stage of disease, presence or absence of comorbidities, and other relevant information, as opposed to ethnicity, which was previously a heavy yardstick for medication choice.

## Introduction and background

Hypertension is a common risk factor for several life-threatening cardiovascular and cerebrovascular diseases, including myocardial infarction, cardiomyopathies, and cerebrovascular accidents [1]. Maintaining optimal blood pressure (BP) control is crucial in managing other significant conditions, including diabetes. Consequently, determining safe cut-off values has remained a primary goal for several organizations, including the American Heart Association (AHA) and the Joint National Committee on Prevention, Detection, Evaluation, and Treatment of High Blood Pressure (JNC). Over the past years, these organizations have focused on developing guidelines to enable physicians and patients to maintain optimal BP control. Depending on the etiology and disease process, several approaches have been recommended to achieve desired BP goals, including lifestyle modifications and medications. However, with evolving knowledge and better understanding, changes in the definition of hypertension, and guidelines to maintain good BP control, have occurred over the decades. These changes have been influenced by factors such as a better understanding of disease pathophysiology, genetics, racial variations, and patient responses to various control modalities [1].

Despite these recommendations, achieving BP control has remained a major concern for healthcare providers and patients. Hence, these guidelines have continued to evolve to accommodate this ever-growing knowledge. This has made keeping up with these organizations' recommendations difficult [1]. This review collects significant guideline reviews on hypertension over the last 22 years and discusses it in a single manuscript. It provides an up-to-date understanding of the concept of hypertension.

Background

According to the 2015 guidelines from the United States Preventive Services Task Force (USPSTF) [1], there is firm evidence to support the control of hypertension as it reduces significant health outcomes, for example, cardiovascular diseases and strokes [1]. Studies show the efficacy of treating the general population aged 60 years and above with specific comorbidities to target BP of less than 150/90 mmHg to decrease the incidence of adverse events such as strokes, cardiovascular events, and heart failure. Trials also showed efficacy in treating younger patients to target diastolic BP less than 90mmHg.The suggested systolic BP is 140 mmHg in adults younger than 60 years, even though this group has documented no benefit for stricter systolic goals. A similar guideline is recommended for hypertensive patients with diabetes or chronic kidney disease to maintain a BP goal of 140/90 mmHg [2].

The 2003 World Health Organization (WHO) and the International Society of Hypertension (ISH) guideline BP reference was systolic BP of 140 mmHg in low-risk patients and lower thresholds in higher-risk patients. The 2014 American Society of Hypertension/International Society of Hypertension guideline goal for diagnosing hypertension was systolic BP > or =140, diastolic BP > 90, or both. This BP goal was based on major clinical trials showing the benefits of treating people with these BP levels [3]. The 2020 international society of hypertension guideline goal is a systolic BP ≥140 mmHg and diastolic BP ≥ 90 mmHg after repeated examinations. This BP diagnostic target was set by extracting evidence-based content from most major guidelines.

The Joint National Commission 8 (JNC 8) guidelines also include treatment recommendations for different age groups, races, and disease conditions [2]. Thiazides or calcium channel blockers have been recommended as first-line pharmacotherapy in the Black hypertensive population. For non-Black populations, thiazides, calcium channel blockers, angiotensin-converting enzyme inhibitors, or angiotensin receptor blockers [1]. Patients with chronic kidney disease or diabetes were also shown to benefit more from an angiotensin-converting enzyme inhibitor or an angiotensin receptor blocker [2]. The 2021 USPSTF guidelines uphold the JNC 8 guidelines on managing hypertension based on the severity of BP, age, and other risk factors for stroke, heart failure, and coronary heart events [3]. These different guidelines aimed to provide a standard of care that would be easy to use in low- and high-resource settings. However, some studies that influenced the set cut-offs for the majority of these guidelines had limitations in that they had insufficient information on younger patients of 18-35 years to determine if there is some benefit in defining hypertension at a lower level and treating them more aggressively than older patients [3]. 

Methodology

We searched four databases: PubMed, Embase, Google Scholar, and Cochrane, using specified search terms. Our keywords were "hypertension" and "guidelines." We combined the keywords using the Boolean operators (AND, OR). We searched for articles written from 2000 to 2022.

A total of 2461 publications were initially identified; 348 publications were removed after screening for full-text availability. The full-text articles were further filtered based on title and abstract screening. A total of 1443 articles were excluded. The remaining 670 full-text articles were assessed for eligibility. Of the 670 full-text articles, 480 were excluded based on exclusion criteria, and following the full-text article screening, 190 articles met the final inclusion criteria.

Inclusion and Exclusion Criteria

1. The reviewed hypertension guidelines that were written between 2000 to 2022: The guidelines documents reviewed were: (a) Amaerican College of Cardiology (ACC)/AHA hypertension guidelines, (b) JNC guidelines, (c) USPSTF recommendations for hypertension, (d) the National Kidney Foundation hypertension guidelines, (e) ISH guidelines, (f) The American Diabetes Association (ADA) guidelines, (g) the European Society of Hypertension (ESH) guidelines.

2. Publication with full-text access indexed in PubMed, EMBASE, Google Scholar, and Cochrane.

We excluded systematic reviews and meta-analyses from this review.

## Review

The ACC/AHA guidelines

The Threshold for Defining Hypertension

The AHA/ACC started synthesizing evidence and publishing guidelines specific to hypertension in 2013. The AHA/ACC hypertension guidelines were initially done in collaboration with the Centers for Disease Control and Prevention (CDC). The AHA/ACC/CDC, in 2013, set a BP goal of 139/89 mmHg for all adults and revised it in 2014 to a screening threshold of 140/90mmHg for all adults [4]. In 2015, the AHA/ACC screening threshold for hypertension for all adults remained the same as the previous year, i.e., 140/90mmHg, but there was a variation in threshold regarding the elderly population, adults with coronary artery disease (CAD), and post-stroke adults. For adults aged > 80 years, the BP goal was <150/90 mmHg; for adults with CAD, it was <140/90mmHg; and for post-stroke adults, it was <130/80mmHg [4]. The 2017 guideline was updated from the seventh report of the JNC (JNC 7), published by the National Heart Lung and Blood Institute (NHLBI) in 2003 [5]. The guideline utilized a broad range of evidence informing the recommendations, including randomized clinical trials, meta-analyses, epidemiological investigations, simulation studies, epidemiological studies, and, in selected cases, expert opinion, to provide an evidence-based approach to lower the risk of cardiovascular disease (CVD) through BP control. The recommendations were stratified by the strength of the recommendation as well as the level of evidence [6,7]. 

The 2017 ACC/AHA guideline eliminated the classification of prehypertension and divided hypertension into three levels: (1) Elevated BP with a systolic BP between 120 mmHg and 129 mmHg and diastolic BP <80mmHg, and (2) Stage 1 hypertension with a systolic BP between 130 and 139 mmHg, and diastolic BP between 80 and 89mmHg and stage 2 hypertension (systolic BP ≥140 mm Hg OR diastolic BP ≥90 mm Hg). A prominent feature in the 2017 guideline was lowering the definition of hypertension from ≥ 140/90mmHg to ≥ 130/80 mmHg [8]. It set a screening threshold of 130/80 mmHg for the diagnosis of hypertension irrespective of age and co-morbid illness status and, as such, advocates for tighter blood pressure control [6,9]. The significantly lower diagnostic threshold (≥130/80 mmHg) beyond that of the previous guidelines ( ≥140/90 mmHg in both the JNC 7 guideline and 2014 evidence-based guideline) was supported by evidence from the Systolic Blood Pressure Intervention Trial (SPRINT), which demonstrated a reduction in cardiovascular events (5.2% vs. 6.8%, hazard ratio (HR) 0.75, 95%CI 0.64-0.89; p < 0.0001) and all-cause mortality (3.3% vs. 4.5%, p = 0.0003) at lower cutoff (120/80) compared to the 140/90 mmHg in the previous guidelines [10]. The 2017 ACC/AHA guideline also recommended a screening threshold of 130/80 for non-institutionalized, ambulatory, community-dwelling adults ≥65 years of age [6]. This was supported by data from the SPRINT trial, in which patients aged ≥75 years (n = 2,636), primary outcomes for intensive vs. routine BP management were 7.7% vs. 11.2%, p < 0.05 and all-cause mortality was 0.73(0.60-0.90) p < 0.05. They also set a screening threshold of 130/80 mmHg for heart failure (optimal BP for those with hypertension), chronic kidney disease, diabetes mellitus, and post-stroke [5]. Whereas evidence from the trial supports a decrease in CVD in chronic kidney disease patients using the recent threshold, it does not demonstrate any benefit for decreasing the progression of renal decline [10]. In the population with heart failure and reduced ejection fraction, ACC/AHA guidelines recommend a threshold of 130/80 mmHg to reduce mortality and heart failure hospitalizations [11]. Whereas the SPRINT study did not include diabetic patients, the primary results for the Action to Control Risk in Diabetes (ACCORD) blood pressure trial only reported a reduction in the risk of stroke (HR, 0.59; 95%CI, 0.39-0.89) at systolic BP < 120 mmHg. However, a re-analysis of the ACCORD trial found that participants randomized to less-intense glycemic control (HbA1c 7.0-7.9%) benefited from targeting systolic BP < 120 mmHg vs. 140 mmHg [10,12].

The ACC/AHA guidelines also pay great attention to accurate BP measurement by recommending standard measurement conditions using appropriately sized BP cuffs and validated BP measurement devices. It also provides new recommendations for out-of-office blood pressure measurement and confirms the diagnosis of hypertension [7].

The Chronology/Changes in Recommendations

In November 2013, the ACC/AHA/CDC collaboration made the following recommendations: ● In stage 1 hypertension, the treatment threshold for adults (systolic BP 140-159 mm Hg or diastolic BP 90-99 mm Hg) may be treated with lifestyle modifications and, if needed, a thiazide diuretic. ● Stage 2 hypertension (systolic BP > 160 mmHg or diastolic BP > 100 mmHg): can be treated with a thiazide diuretic combined with an ACEI, an ARB, or a calcium channel blocker. The recommended BP goal should be 139/89 mmHg or less. ● For individuals who do not attain BP goals, medication dosing may be increased, or medication with a different mechanism of action can be added to the treatment [4]. ● In 2014, the recommended BP goal in all adults was revised to <140/90mmHg.

The AHA/ACC/ASH collaboration released a guideline on the management of hypertension in patients with coronary artery disease in 2015 and made the following recommendations: ● A BP control of <140/90 mm Hg was recommended for most CAD and hypertension patients. ● A BP of 130/80 mm Hg was reasonable in individuals with CAD, its equivalent, and stroke. ● In individuals with hypertension and CAD, a BP target of <140/90 mm Hg for secondary prevention of cardiovascular events seemed reasonable, and tighter control of 130/80 mmHg was acceptable in some of these patients with a previous stroke, transient ischemic attack, myocardial infarction (MI), or CAD risk equivalents (CAD, peripheral artery disease, abdominal aortic aneurysm). ● Medications recommended for patients with hypertension and chronic stable angina include ACEIs or ARBs, beta-blockers (history of MI), and thiazide. They also mentioned that medications with mortality benefits in patients with acute coronary syndrome (ACS) were beta-blockers, ACEIs or ARBs, and possibly aldosterone antagonists. ● In patients with CAD with elevated diastolic BP and myocardial ischemia, BP reduction should be gradual. Caution should be taken when lowering diastolic BP to less than 60 mmHg in the elderly and individuals with diabetes mellitus. Lowering systolic BP in older patients with hypertension and wide pulse pressures may reduce diastolic BP (less than 60 mmHg) [13]. ● In 2017, the ACC/AHA guideline was a comprehensive guideline incorporating new information from studies regarding BP, the related risk of CVD, ambulatory BP monitoring, home BP monitoring, BP thresholds to initiate antihypertensive drug treatment, BP goals of treatment, strategies to improve hypertension treatment and control, and various other vital issues. In Table 1, an overview of the BP threshold and management therapy goals are given [5].

**Table 1:** Blood pressure thresholds and goals of pharmacologic therapy in patients with hypertension according to clinical conditions CVD: cardiovascular disease; ASCVD: atherosclerotic cardiovascular disease; BP: blood pressure

Clinical Condition(s)	BP Threshold mmHg	BP Goal mmHg		
			General				
Clinical CVD or 10-year ASCVD risk ≥ 10%	≥130/80	<130/80					
			No clinical CVD and 10-year ASCVD risk <10%	≥140/90	<130/80		
Older persons (≥65 years of age; non-institutionalized, ambulatory, community-living adults older persons (≥65 years of age; non-institutionalized, ambulatory, community-living adults)	≥130 (SBP)	<130 (SBP)					
			Special comorbidities				
Diabetes mellitus	≥130/80	<130/80					
			Chronic kidney disease	≥130/80	<130/80		
Chronic kidney disease post-renal transplantation	≥130/80	<130/80					
			Heart failure	≥130/80	<130/80		
Stable ischemic heart disease	≥130/80	<130/80					
			Secondary stroke prevention	≥140/90	<130/80		
Peripheral arterial disease	≥130/80	<130/80					

Limitations to ACC/AHA Considerations

The SPRINT and ACCORD clinical trials were two significant studies that shaped the most recent ACC/AHA guidelines. They, however, had certain limitations that may have affected their results: (1) The SPRINT trial showed a better cardiovascular outcome among the treatment group that received intensive treatment (aiming for systolic BP < 120 mmHg) compared with a standard treatment group (systolic BP < 140 mmHg), excluded individuals with end-stage chronic kidney disease (glomerular filtration rate < 20 mL/min/m^2^ and proteinuria > 1 g/day), diabetes, and population in nursing homes or had dementia resulting in lack of generalizability (external validity) [14], and (2) The ACCORD BP trial revealed that tight BP control showed no advantage over standard therapy. However, many scholars felt the study was "underpowered" because of its design [14]. 

All analyzed evidence favours a lower achieved blood pressure for many patients. This lower goal may be challenging for a handful, particularly in elderly individuals with vascular stiffness. This population also tends to have low diastolic pressure. Reducing diastolic BP below 60 mm Hg in those with CAD may magnify the risk of adverse cardiovascular events. The guidelines failed to address the issues of lowering diastolic BP [14].

ESC (European Society of Cardiology)/ESH Guidelines

The Threshold for Defining Hypertension

Before 2013, the ESH and the ESC did not have a particular guideline on hypertension, as they were working with and endorsed guidelines of the ISH Liaison Committee/World Health Organization (WHO) [15,16]. They added these guidelines, with some modifications, to the joint European recommendations for preventing coronary heart disease [17,18]. Previously, diastolic BP was emphasized over systolic BP as a predictor of cerebrovascular and coronary heart disease. This was reflected as diastolic BP being the primary inclusion criterion for randomized control trials [19]. However, both systolic and diastolic BPs showed a continuous graded independent relationship with the risk of stroke and coronary events [20], demonstrating the significance of using both systolic and diastolic BP to guide treatment thresholds [21].

The treatment intervention levels for cardiovascular risk factors such as BP, blood cholesterol, and blood sugar have been based on different arbitrary points of the individual risk factors; the current approach to management is to determine the threshold, at least for cholesterol and BP reduction, based on the calculation of estimated coronary risk over a defined period (e.g., five years or 10 years) [17,18]. In this guideline, the risk estimation systems are based on Framingham data and the SCORE project, which provides a table that predicts the 10-year risk of CVD separately for higher-risk countries in northern Europe and lower-risk countries in southern Europe [22]. The BP classification given in Table 2 was used [23].

**Table 2:** ESC/ESH blood pressure classification of hypertension from 2003 was retained in 2007 and 2018 but came with some clauses in 2007 and is currently in use today ESC: European Society of Cardiology; ESH: European Society of Hypertension

CATEGORY	SYSTOLIC	DIASTOLIC
Optimal	120	80
Normal	120–129	80–84
High normal	130–139	85–89
Grade 1 hypertension (mild)	140–159	90–99
Grade 2 hypertension (moderate)	160–179	100–109
Grade 3 hypertension (severe)	> 180	> 110
Isolated systolic hypertension	> 140	90

The Chronology/Changes in Recommendations

The significant chronologic changes in the ESC/ESH guidelines for treating hypertension from 2001 to 2021 were in the areas of the threshold for drug initiation and target BP. The choice of drug classes effective in treating hypertension and the combination recommended for hypertensives with various comorbidities remained constant.

2003 ESC/ESH Treatment Guidelines: The 2003 guidelines and recommendations on the threshold for the initiation of drug therapy include the following: Irrespective of the patient’s age, the decision to initiate antihypertensive therapy is governed by cardiovascular risk and the level of systolic and diastolic BP, with cardiovascular risk-taking precedence over BP [21]. They recommended drug treatment when a patient has had high-normal BP on several occasions with high or very high cardiovascular risk factors. Patients with moderate cardiovascular risk should be monitored frequently, and no interventions were needed for low-risk individuals. For grade 1 and grade 2 hypertension, in addition to high and very high-risk patients, those with moderate risk are to commence drug treatment if they continue to have systolic BP ≥140 mmHg or diastolic BP ≥90 mmHg after three months of monitoring BP and other risk factors [21]. Also, drug treatment can be considered if the low-risk patient continues to have systolic BP ≥150-159 mmHg or diastolic BP ≥90-99 mmHg and the patient prefers it [21]. Patients with grade 3 hypertension are to begin treatment immediately.

Concerning special conditions such as patient's age and comorbidities (diabetes mellitus, chronic kidney disease, post-stroke, hypersensitivity lung disease, heart failure): ● The initiation of antihypertensives should follow the general guidelines while paying attention to the patient’s risk factors and frailty, target organ damage, and associated cardiovascular conditions, irrespective of the patient’s age [21]. ● In diabetic patients, lifestyle modification is recommended in all type 2 diabetics, irrespective of BP. This may be enough to normalize BP in some cases of high normal and grade 1 BP. It can enhance BP control in those on antihypertensives. While the ESC/ESH did not delineate the exact BP levels for the initiation of drug treatment, antihypertensives were recommended for all patients with grade 1 or 2 hypertension who have microalbuminuria [21]. ● For the other comorbid conditions (CKD, post-stroke, HLD, heart failure), there were no clear guidelines on initiating antihypertensives different from the ones stated above.

The committee recommended that BP be lowered to ≤140/90mmHg in all patients and <130/80mmHg in people with diabetes while acknowledging that it may be challenging to achieve SBP <140mmHg in elderly patients [21]. The 2003 guidelines and recommendations on the use of antihypertensives and choice of therapy were: ● Drug treatment should be gradual and low, allowing several weeks to reach the target BP level. ● Patients are more likely to reach the target BP with the combination of more agents than with just one agent. ● It emphasized the need for lower BP over the choice of agent to achieve cardiovascular benefit. Also, more emphasis was placed on using a combination of two or more drugs to achieve the desired BP target rather than identifying the first class of drug to use [21]. ● It recommended diuretics, β-blockers, calcium antagonists, ACEIs, and ARBs as appropriate agents for the initiation and maintenance of treatment [21]. ● The committee recommended once-daily preparation with 24 hours efficacy where feasible.

Based on evidence from interventional studies, we found the following combinations to be beneficial: Diuretics plus ACE inhibitors or angiotensin II receptor blockers (ARBs), β-blockers, or calcium antagonists, ACEIs or ARBs plus calcium antagonists, β-blockers plus calcium antagonists [21]. While most of the studies reviewed by the committee demonstrated clear benefits with BP reduction in post-stroke patients who were both normotensive and hypertensive, the ESC/ESH did not recommend any agent for this group of patients. The committee recommended that BP be lowered to ≤140/90 mmHg in all patients and <130/80 mmHg in people with diabetes while acknowledging that it may be challenging to achieve SBP <140 mmHg in elderly patients [21].

The 2007 ESC/ESH Treatment Guidelines

Based on more recent studies after the 2003 guidelines were published, the ESC/ESH published a new set of guidelines in 2007 while still adhering to most of the recommendations in the 2003 guidelines. The ESC/ESH committee made the following recommendations concerning the initiation of antihypertensive therapy: ● Again, the decision to initiate antihypertensives is governed by the presence of cardiovascular risk and the level of systolic and diastolic BP. ● The ESC/ESH guidelines used the 10-year risk of having a cardiovascular event (fatal or non-fatal) to stratify risk into low, moderate, high, and very high risk. But an additional term "added risk"-was used to imply that the risk was higher than average in all strata [23]. ● In the 2007 guidelines, the immediate initiation of treatment for all patients with grade 3 hypertension and very high risk (even in patients with normal BP) with therapy and lifestyle modification was recommended [23]. ● They also recommended treating patients with high-normal BP and grade 1 and 2 BP with high risk and lifestyle modification. ● Grade 1 and 2 patients were to begin treatment only after several months of uncontrolled BP with lifestyle modifications.

The 2007 guidelines and recommendations on using antihypertensives, choice, and treatment targets or goals were still the same as the 2003 guidelines in these areas. The notable changes were: The need to use peak and trough or ambulatory measurements to ensure 24 hours of BP monitoring. Also, in addition to calcium antagonists and thiazide diuretics, ACEIs and beta-blockers were recommended to treat hypertension in elderly patients. The ESC/ESH recommended a tighter BP target goal of 130/80 mmHg for patients with previous stroke and renal dysfunction (and even lower for patients who had > 1 g/day of protein in their urine) [23]. They recommended a fixed-dose combination to ensure compliance

2013 ESC/ESH Treatment Guidelines

In the 2013 guidelines, the committee made some reversals in their earlier decisions to initiate drugs and treatment targets in elderly patients and patients with comorbidities like diabetes mellitus and post-stroke.

The recommendations for the initiation of drug treatment in the 2013 guidelines are: ● Based on new evidence, the committee recommended no intervention for patients with high normal BP compared to the previous guidelines, as most of the studies in support of intervention either classified patients already on antihypertensives as normotensive or had a tiny percentage of normotensive patients in their research [24]. ● It recommended intervention for the elderly when the systolic BP is >160 mmHg as this is the level at which intervention supports the result in elderly patients [24]. ● For youths with isolated systolic BP >140 mmHg, it recommended close monitoring as evidence did not support these patients' systolic/diastolic BP progression.

In the area of target BP, based on evidence from recent studies [24-26], the 2013 committee recommended systolic BP of <140 mmHg for all patients irrespective of comorbidity, including elderly patients <80 years of age. For elderly patients older than 80 years, a systolic BP reduction of between 150-140 mmHg was recommended. A diastolic BP of <90 mmHg was adopted in all patients, except in people with diabetes, who were found to benefit from a diastolic BP reduction of between 85-80 mmHg but not less than 80 mmHg. In terms of therapeutic strategies and choice of drugs, the committee mainly reaffirmed the position of the earlier guidelines in areas such as the use of combination therapy, fixed-dose formulation, significant classes of antihypertensives suitable for initiation of treatment, and the choice of drugs for comorbid conditions. It recommended therapy for youths with diastolic BP >90 mmHg. In the 2013 guidelines, the committee looked at chronic kidney disease patients separately from other nephropathies and recommended all antidiabetics except diuretics.

2018 ESC/ESH Treatment Recommendations

In 2018, the ESC/ESH committee made important changes based on new evidence from studies regarding when drug treatment should be initiated, target BP, and changes in the management of hypertension in patients with comorbidities and across the age spectrum.

The Start of Therapy: The 2018 committee recommendations on drug treatment initiation for the different hypertension grades are shown in Table 3. For all non-frail elderly patients, initiate treatment when systolic BP is ≥160mmHg. Treatment should also be undertaken for fit, elderly patients with systolic BP of 140-149mmHg [27]. The committee discouraged the discontinuation of therapy in fit patients based on age when treatment is tolerated. Treatment should also be initiated for patients with chronic kidney disease, diabetes mellitus, and a previous stroke who have office systolic BP ≥140 mmHg or office diastolic BP ≥90 mmHg [27].

**Table 3:** ESC/ESH treatment thresholds ESC: European Society of Cardiology; ESH: European Society of Hypertension; BP: blood pressure

BP grades	High normal	Grade 1	Grade 2	Grade 3
Recommendation	May intervene in high-risk patients with cardiac pathology.	Initiate treatment for high or very high with no organ damage, cardiovascular disease, or kidney disease. Low or moderate risk with organ damage, cardiac or renal pathology: initiate drug treatment after three to six months of monitoring	Initiate treatment for all patients	Initiate treatment for all patients

Treatment Objectives: The committee recommended the following treatment targets ● To keep the office BP of these patients to <140mmHg office SBP and <90mmHg if tolerated. ● When tolerated, SBP should be between 120 - 129mmHg for patients < 65 years. ● 130 -139mmHg was recommended for patients >65 years ● It advised diabetic patients to keep their blood pressure below 130 mmHg. ● Target DBP for all patients should be <80mmHg

Therapeutic strategies, drug choice, and other related therapeutic guidelines were unchanged. 

Research Trials Considered in the ESC/ESH Guidelines and Their Limitations

The SCOPE study, cardiology vascular health study, atherosclerosis risk in a community study, the Hypertension Optimal Treatment (HOT) study, Australian blood pressure study, Antihypertensive and Lipid-Lowering Treatment to Prevent Heart Attack Trial (ALLHAT), Lifestyle Interventions and Independence for Elders (LIFE) study, ASCOT study, SPRINT, and the Prevention and Treatment of Hypertension study [21,23,27] are some of the research studies and trials that were used in making decisions for these guidelines. The short-duration controlled randomized trials lasted for four to five years, whereas life expectancy in middle-aged hypertensives is 20-30 years. Furthermore, exclusion/selection criteria for patients most often at elevated cardiovascular risk and extrapolation to patients at a different risk level is doubtful. Meta-analyses don't necessarily represent the highest level of evidence. However, they have greater statistical power than individual trials and may provide helpful average measurements of treatment effects. They are post hoc analyses. The trials included are not homogeneous, with differences not always susceptible to being assessed by statistical tests. Most trials are not powered for secondary endpoints. Therapeutic programs in trials often diverge from those followed in clinical practice, and patient compliance in trials is much higher than in clinical practice.

The USPSTF guidelines

The USPSTF is an independent, volunteer panel of national disease prevention and evidence-based medicine experts. The task force primarily makes evidence-based recommendations about disease prevention. Regarding hypertension, most of the USPSTF work was focused on screening strategies.

In 1996, the USPSTF recommended only screening adults (21 years or older) for hypertension. At this time, no treatment guidelines/recommendations for hypertension were made by the task force [28]. In 2003, the USPSTF updated its hypertension guidelines to screen adults aged 18 and above. In this update, the USPSTF commented on a systematic review of eight trials that showed that treating isolated systolic hypertension in the elderly significantly reduced coronary artery disease and stroke rates [29]. The same study found a decreased mortality in this group by 13%, with number needed to treat 18 (for men) and 38 (for women) over five years. The USPSTF also commented that diabetic patients derived additional benefits from more aggressive BP control (less than 140/90 mmHg) [29]. These comments were made based on findings derived from studies such as the United Kingdom Prospective Diabetes Study (UKPDS) and the HOT Trial [29]. In the UKPDS, fewer events of any diabetes-related clinical endpoint and fewer diabetes-related deaths were experienced in the more aggressive BP control arm compared with, the less aggressive arm. In the HOT trial, it was observed that more aggressive BP treatment in diabetic patients reduced major cardiovascular events by 49% [29]. There was a paucity of evidence for the task force to recommend more aggressive BP control in the general population. Furthermore, the USPSTF did not have evidence to suggest nonpharmacologic therapies for managing hypertension [29].

The USPSTF in 2007 sought to ask two questions: (1) What are the benefits of screening for high BP in adults? (2) What are the risks of early screening for and/or treating high BP? A literature search on studies on the benefits and harms of screening and treatment of hypertension was conducted by the task force. They included studies of nonpregnant adults in the United States and outside of the United States with a patient population generalizable to the United States. To meet their inclusion criteria, the USPSTF found no studies on the benefits or harms of screening for high BP. Five studies on the harms of early hypertension treatment met their inclusion criteria [30]. In these studies, the USPSTF noticed common side effects of pharmacotherapy, but severe adverse effects were rare. Based on the above evidence, the task force concluded that the benefits of early screening and treatment of hypertension were more significant than the risk associated with minor side effects of pharmacotherapy. The USPSTF commented that non-pharmacology (lifestyle) therapies such as reducing dietary sodium intake, increasing physical activity, weight loss, decreasing alcohol intake, and stress management are associated with lowering BP. This was an update compared to 2003 when the task force did not find substantial evidence to comment on nonpharmacologic therapies for BP management [30].

In 2015, the USPSTF reaffirmed its recommendation to screen for high BP in adults older than 18. The task force also recommended that caregivers obtain BP measurements from outside the clinic to confirm the diagnosis of high BP before initiating treatment [31]. This was the first time the task force recommended ambulatory BP measurement as a diagnostic tool. Based on the results of multiple randomized control trials, the task force in 2015 recommended that the general population aged 60 and older be treated to a target BP of 150/90 mmHg to achieve a reduced incidence of stroke, heart failure, and coronary heart disease events. Based on evidence from randomized control trials, the USPSTF recommended a target diastolic BP of <90 mmHg to reduce cerebrovascular events, heart failure, and overall mortality based on evidence from randomized control trials [31]. But there was insufficient randomized clinical trial data to recommend a systolic BP goal of less than 140 mmHg in adults younger than 60; thus, the USPSTF made this recommendation in 2015 based on expert opinion [32].

USPSTF also commented on first-line pharmacological therapy for different groups of patients. For non-Black patients, the initial treatment recommendation consisted of a thiazide diuretic, calcium-channel blocker, angiotensin-converting enzyme inhibitor, or angiotensin-receptor blocker. A thiazide diuretic or a calcium-channel blocker was recommended for Black/African American patients as the first-line treatment [31]. For patients with chronic kidney disease, an ACEI or an ARB (not both) was recommended as first-line or add-on therapy. Notably, the task force did not provide any evidence or rationale for the above recommendations [31]. In their 2021 guidelines, the USPSTF reaffirmed its 2015 recommendation to screen all adults (18 years and older) for hypertension using office BP measurement. They continued to recommend obtaining BP measurements outside of the clinical setting to confirm the diagnosis of hypertension before treatment initiation [33].

The NKF

In 1998, the NKF published the task force's report on CVD in chronic renal diseases. One of the objectives was to assess current knowledge about the association between high BP and CVD in chronic kidney disease. Also, the NKF published a report on managing hypertension in adults with diabetes and renal disease. In 2001, the NKF initiated a Kidney Disease Outcomes Quality Initiative (KDOQI) workgroup to conduct a review of evidence and develop clinical practice guidelines for the management of blood pressure in chronic kidney disease to prevent the progression of kidney disease and the development and progression of CVD in chronic kidney disease. The goal of this guideline was to relate high BP to adverse outcomes of chronic kidney disease and to describe the association of the level of glomerular filtration rate with high BP. 

Most of the time, they made decisions based on their findings through workgroups and Kidney International (kidney disease; Improving Global Outcomes), if done in collaboration, from clinical trials and observational studies based on risk stratification. Some of their guidelines on managing hypertension in diabetic chronic kidney disease were consistent with the American Diabetes Association and JNC data. In addition, KDOQI used the glomerular filtration rate as the principal basis for staging chronic kidney disease and augmented the staging scheme by incorporating the cause of kidney disease and level of albuminuria in addition to the level of glomerular filtration rate. 

Many clinical trials on antihypertensive agents to prevent CVD excluded patients with reduced kidney function. In 2007 guidelines, Type 1 and 2 diabetes mellitus and chronic kidney disease stages 1-4 were included, while kidney transplant recipients were excluded; Table 4 is a chronology of the various recommendations over time.

**Table 4:** The chronology of the changes in guidelines ACEI: angiotensin-converting enzyme inhibitor; ARB: angiotensin receptor blocker; CKD: chronic kidney disease; BP: blood pressure

Year	Comorbidity	Changes made
2002	CKD	The recommended goal of antihypertensive therapy for patients at low or moderate risk for complications is to maintain systolic and diastolic BP less than 140- and 90-mm Hg, respectively.; Target BP is lower in younger patients and is related to age, weight, and height.; Target BP should be <130/85 mm hg for individuals with high blood pressure and decreased kidney function; In the general population, the recommended antihypertensive agents are diuretics and beta-adrenergic blockers. There should also be reduction in dietary salt and regular exercise; There is equal efficacy of ACEIs and calcium channel blockers in the general population
2002	Diabetic kidney disease or CKD stages 1-4 with proteinuria(>1g/dl); CKD stages 1-4 without proteinuria; CKD stage 5	Alternative target blood pressure and medications may be preferred in those subgroups of patients with comorbid conditions; In previous years, the target BP was 140/90 mmHg, especially in type one diabetes mellitus but BP of <125/75 has been recommended, alongside reduction in dietary salt; Use of ACEIs or ARBs and diuretics; For CKD stages 1-4 without proteinuria (<1 g/dl), Target BP is <135/85. There should also be a reduction in dietary salt intake and use of ACEI or ARBs; For CKD stage 5, Target BP is <140/50. There should be a reduction in dietary salt, fluid intake, and ultrafiltration in dialysis patients.
	Nondiabetic kidney diseases	No modifications were noted, although the NKF Task Force recommended target blood pressure levels and strategies for treatment for patients with CKD; ACEIs were recommended.
	Kidney transplant recipients	Use of calcium channel blockers for kidney transplant recipients.
2007 (Guideline 7)	CKD	Target BP for cardiovascular risk reduction should be <130/80 mmHg: Antihypertensives used depend on other underlying pathologies, if present.; Diuretics are also included in the regimen of most patients.
	Heart failure with diastolic dysfunction	Thiazide or loop diuretics; ACEIs or ARBs; aldosterone antagonists; beta-blockers.
	Post myocardial infarction with systolic dysfunction	ACEIs or ARBs; beta-blockers; aldosterone antagonists
	Post myocardial Infarction	Beta-blockers
	Recurrent stroke prevention	Thiazide or loop diuretics; ACEIs or ARBs
	Supraventricular tachycardia	Beta-blockers; calcium channel blockers
2004 (Guideline 8)	Diabetes in kidney disease	Target BP-<130/90 mmHg; Patients with or without hypertension should be treated with ACEIs or ARBs.
2004 (Guideline 9)	Nondiabetic kidney disease	Target BP -<130/90 mmHg • Patients with or without hypertension should be treated with ACEIs or ARBs.
2004 (Guideline 10)	Kidney disease in the kidney transplant patient	Calcium channel blockers, diuretics, ACEIs, ARBs, beta-blockers
2004 (Guideline 11)	CKD	ACEIs and ARBs can be used safely in most patients with CKD; ACEIs and ARBs should be used in moderate to high doses, as used in clinical trials; They should be used as alternatives to each other if the preferred class cannot be used; They can be used in combination to lower BP or to reduce proteinuria; Patients treated with ACEIs should be monitored for hypotension, decreased GFR, and hyperkalemia; ACEIs or ARBs can be continued if GFR decline over four months is <30% from the baseline and/or serum potassium level is = 5.5 mEq/L
2005	CKD (hypertension control in the dialysis patient)	Pre-dialysis BP goals- <140/90 mmHg; Post dialysis BP goals- <130/80 mmHg.; Use of renin-angiotensin inhibitors such as ACEIs or ARBs
	BP control in children	Optimal systolic and diastolic BP should be < 95% for age, gender and height; Management of BP pays attention here to fluid status and antihypertensive medications and minimizing intradialytic fluid accumulation; Education by dieticians every three months; Low salt intake; Longer dialysis ultrafiltration
2007	Diabetes and CKD (stages 1-4)	Target BP in diabetes and CKD stages 1-4 should be <130/80mmhg; Hypertensive people with diabetes and CKD stages 1-4 should be treated with an ACEI or an ARB, usually in combination with a diuretic(preferred); then a beta-blocker or calcium channel blocker.
2012	CKD (in relation to age, nondiabetic kidney disease, diabetic kidney disease, kidney transplant recipients	For patients with CKD with normal to mildly increased albuminuria, goal BP has been relaxed to =140/90 mm hg for both diabetic and nondiabetic patients.. However, target BP for patients with CKD with moderately or severely increased albuminuria and all renal transplant patients with/without the presence of proteinuria should be = 130/80 mmHg.
	Age	For BP control in the elderly, a BP goal of <140/90 mmHg is acceptable, and the choices are not mandated In adults with diabetes in CKD, and with mild albuminuria, they should be maintained at a bp of < or =140 mmHg, and a diastolic of < or =90 mmHg. In adults with diabetes in CKD, and with moderate to severe albuminuria, they should be maintained at a bp of < or =130 mmHg, and a diastolic of < or =80 mmHg

Chronology of the Changes in the NKF Guidelines

There were a number of research trials in the NKF guidelines, each with its limitations.

2002 Clinical Practice Guidelines for Chronic Kidney Disease: Evaluation, Classification, and Stratification: In 2002, according to the NKF guidelines for chronic kidney disease, the recommendations for the general population were based on evidence from observational studies and clinical trials that related BP levels to mortality and CVD. This was seen in the HOT on diabetic kidney disease [34]. There was also a general agreement that risk stratification was used to decide which patients with high BP should be treated and the intensiveness of a treatment plan. This is why it seemed reasonable to recommend lower target BP levels for patients with diabetic kidney disease. Furthermore, ACEIs and ARBs have been shown to reduce glomerular capillary blood pressure and protein filtration, which may result in a slower progression of the disease in diabetic and nondiabetic kidney disease. The Heart Outcomes Prevention Evaluation (HOPE) study revealed reduced mortality benefits for nondiabetics with normal renal function [34].

2004 Hypertension and Hypertensive Agents in Chronic Kidney Disease (Guideline 7,8,9): Numerous epidemiological studies showed a graded, independent, and strong relationship between arterial BP and CVD. Above a systolic BP of 115 mmHg and a diastolic BP of 75 mmHg, there is a risk of CVD, which double for every increment of the systolic BP by 20 mmHg or diastolic BP by 10 mmHg. In individuals above 50 years of age, systolic BP greater than 140 mmHg is a more critical CVD risk factor than diastolic BP [35]. Controlled trials and observational studies also showed a need to reduce BP levels in diabetic kidney disease or even heart failure. For each patient, the clinician determined if there was an indication for a preferred agent based on the type of chronic kidney disease, co-existing CVD, and other comorbid conditions and defined the therapeutic goals for each indication. In persons with more than one indication, treatment decisions were to be individualized according to the risk for CKD progression, reducing the risk of CVD or other outcomes [35].

The onset of hypertension in persons with type 1 diabetes usually signifies the beginning of renal disease. In contrast, in those with type 2 diabetes, it may occur without kidney disease(Guideline 8). Several prospective studies have shown a strong correlation between BP and an increased risk of renal failure in diabetic kidney disease [35].

Generally, multiple antihypertensive agents are required to reach target BP levels. Nevertheless, ACEIs are more effective than other antihypertensive classes in slowing the progression of kidney disease with macro-albuminuria due to type 1 diabetes. Also, ARBs are more effective than other antihypertensive classes in slowing the progression of kidney disease with macro-albuminuria due to type 2 diabetes [35].

2005 KDOQI Clinical Practice Guidelines for Cardiovascular Disease in Dialysis Patients: The rationale for the recommendations was from the reviews that linked hypertensive heart disease to cardiovascular morbidity and mortality [36]. People with diabetes on dialysis may be more prone to postural hypotension and labile BP than nondiabetic dialysis patients. Permissive elevated supine BP may be necessary to prevent symptomatic postural hypotension [36].

2007 American Journal of Kidney Disease, Clinical Practice Guidelines and Clinical Recommendations for Diabetes and Chronic Kidney Disease: The guidelines for antihypertensive agents in kidney disease due to diabetes and other causes do not conflict. ARBs and ACEIs were compared with other classes of antihypertensive agents. These studies frequently used diuretics as additional antihypertensive agents to achieve BP control [37].

2012 KDOQI United States Commentary on the 2012 KDIGO Clinical Practice Guidelines for the Management of BP in Chronic Kidney Disease: Per the National Health and Nutrition Examination Study (NHANES) data, chronic kidney disease and nondiabetic chronic kidney disease are often associated with resistant hypertension. The choice of agents for treatment must be made according to the comorbidities, especially renovascular disease, volume depletion, or potential drug interactions [38].

The concept that diuretics complement ACEIs/ARBs in combination is well supported. While the metabolic side effects of thiazide diuretics are well known, these are usually easily managed. Interest in chlorthalidone was on the rise as the diuretic of choice, as most of the large clinical trials used chlorthalidone. Beta-blockers are still utilized in hypertensive patients with chronic kidney disease. In primary hypertension, beta-blockers were no longer considered first-line therapy in hypertension without indication [38].

For BP control in chronic kidney disease nondiabetics, evidence was scarce for these recommendations that suggested that except in the individuals with severely elevated albuminuria, there was no compelling reason to use or not use specific agent classes in these patients [38].

Based on the Hypertension in the Very Elderly Trial (HyVET), a target BP of 140-145 mmHg was recommended for octogenarians. Because elderly patients with nondiabetic kidney disease may require less aggressive BP reduction, the above-average blood pressure target may apply to these individuals [38].

The Limitations of These Studies

The 2002 Clinical Practice Guidelines for Chronic Kidney Disease: Evaluation, Classification, and Stratification: The 2002 survey done by the NKF showed that the knowledge base for chronic kidney disease was limited. The formulation of this guideline was also limited by other factors, such as the fact that a number of the studies were difficult to compare as they used different measures for kidney function including glomerular filtration rate or creatinine clearance, estimation equations for glomerular filtration rate or creatinine clearance, or simply serum creatinine [34]. Secondly, this guideline failed to provide a semi-quantitative analysis of the relationship between the factors and the outcomes for the rate of progression and risk of kidney failure [34].

The 2004 Hypertension and Hypertensive Agents in Chronic Kidney Disease (Guideline 7,8,9): There were few studies on treating hypertension in chronic kidney disease. Most clinical studies in patients with chronic kidney disease were designed to evaluate the efficacy of therapy in slowing the progression of chronic kidney disease rather than reducing the risk of CVDs [35]. These recommendations need to be qualified based on the available data. First, no claims of superiority between ACEIs and ARBs can be made since no randomized trials have compared these agents "head-to-head" in slowing the progression of kidney disease. Secondly, the efficacy of therapy in many studies of diabetic kidney disease with microalbuminuria, the effectiveness of antihypertensive agents was based on reduced risk of kidney disease progression, as assessed by the development of macro-albuminuria, rather than a decline in glomerular filtration rate or onset of kidney failure [35].

The KDOQI 2005 Guidelines for CVD in Individuals with Renal Impairment on Dialysis: Many of the recommendations supported by the ADA on the care of diabetes are based on extensive clinical studies that provide strong evidence for the particular recommendation, but those studies do not target dialysis patients [36].

The 2007 American Journal of Kidney Disease, Clinical Practice Guidelines and Clinical Recommendations for Diabetes and Chronic Kidney Disease: Combining ACEIs with ARBs effectively slowed the progression of nondiabetic kidney disease, an observation related to a further reduction in proteinuria rather than BP lowering. No trials with clinical outcomes had evaluated such a combination for treating diabetic kidney disease. Other combinations, such as aldosterone blockade with ACE inhibition, may have reduced albuminuria independent of blood pressure changes in diabetic kidney disease. To date, all studies evaluating combinations of renin-angiotensin-system (RAS) inhibitors had been performed in hypertensive patients with diabetes with advanced chronic kidney disease and microalbuminuria [37].

The 2012 KDOQI United States Commentary on the 2012 KDIGO Clinical Practice Guidelines for Managing BP in Chronic Kidney Disease: There has been a lack of evidence for most of the guidelines. One of the more controversial recommendations was to treat patients with chronic kidney disease, with or without diabetes, who have moderately to severely increased albuminuria to a systolic BP of 130 mmHg. Although this may result in less albuminuria than a less stringent goal of 140 mm Hg, it is hard to show the benefit of the 130 mmHg systolic goal, which conflicted with the JNC's recommendations at the time [38].

The ADA guidelines

The "Standards of Medical Care in Diabetes" is a component of the ADA guidelines, which outline the current recommendations for the clinical management of diabetes. These include the components of care for diabetes patients, treatment goals, and tools to assess the quality of care [39]. With the rapid evolution of technology, scientific research, and treatments for diabetes, these guidelines are constantly updated to reflect the latest evidence. Below are the guidelines and treatment threshold changes for diabetes patients regarding age and various comorbidities [39].

Among young people, type 1 diabetes is the most common type of diabetes. Individualized nutritional therapy is recommended for this population as a crucial part of the treatment plan. The recommended pharmacologic therapy for people with type 1 diabetes includes intensive insulin treatments. Diabetes is also prevalent in older adults, especially people over 65 years of age. This population commonly has coexisting chronic illnesses, which should be assessed at the initial visit and annually, as needed [39]. The aim of having treatment goals for diabetes is to eliminate or delay the incidence of complications and improve quality of life. The ADA guidelines advocate a thorough medical evaluation during the first visit to meet the following objectives: confirmation of the diagnosis, classification of diabetes, assessment of complications of diabetes or common comorbidities affecting individuals with diabetes, risk factor control in already diagnosed individuals, and development of a plan of care. The current guideline advises against intensive glucose control in hyperglycemic patients to improve cognitive function in type 2 diabetes mellitus patients [39]. The ADA guidelines outline various recommendations for people with diabetes and CVD. First, BP should be assessed routinely at every clinical visit, and appropriate pharmacologic intervention should be available for BPs greater than 140/90 mmHg. ACEIs or ARBs are the recommended first-line treatment for hypertension in individuals with diabetes and CAD [40]. Secondly, lifestyle modifications, such as dietary approaches to stop hypertension (DASH) or a Mediterranean-style meal plan, are recommended to control hyperlipidemia. Aspirin therapy (75-162 mg/day) is also recommended as a primary prevention strategy for diabetics with an increased cardiovascular risk. Studies show pioglitazone is an effective treatment for type 2 diabetes patients with post-stroke depression [40].

For many years, the ADA has recommended that people with diabetes get their blood glucose tested two to four times a year using an A1C (glycated haemoglobin) test. One of the major highlights of the 2021's guideline update was that sodium-glucose cotransporter-2 (SGLT-2) inhibitors or glucagon-like peptide 1 (GLP-1) agonists (if SLGT-2 is not well tolerated) be used in people with diabetes with comorbid conditions such as chronic kidney disease and heart failure, irrespective of HbA1C or metformin use.

The ADA 2021 guidelines were updated to include the following recommendations: ● Individuals with diabetes and concomitant hypertension at higher cardiovascular risk or 10-year ASCVD risk > 15% are to be treated to a BP target of < 130/80 mmHg, whereas a BP target of < 140/90 mmHg is to be achieved by people with diabetes and hypertension at lower cardiovascular risk or 10-year ASCVD risk < 15% [41]. ● ACEIs and ARBs are now recommended as first-line treatments for hypertension in people with type 2 diabetes and CAD. Also, the recommended BP target during pregnancy was reduced to 110-135/85 mmHg to minimize the risk of developing gestational-induced hypertension [41]. ● Time in Range (TIR) is a valuable metric in diabetes care and was set to be above 70%, and the Time Below Range (TBR) is lower than 4% for most adults with an A1C below 7% and has been proven to improve diabetes management. The changes were made to achieve a target range (70-180 mg/dl) more than 70% of the time, with less than 4% of the time spent below the range (under 70 mg/dl) [41]. ● Continuous glucose monitoring for people with diabetes on multiple insulin injections or an insulin pump was recommended to improve day-to-day glycemic control and achieve positive long-term health outcomes. Previously, continuous glucose monitoring was only used in people who did not meet the glucose targets or had hypoglycemic episodes [41]. ● Assessment of social determinants in population health, medical evaluation, the care of youths, and obesity management at the initial and annual diabetes evaluation visits ● An emphasis was made on the need for healthcare professionals to use the 2020 diabetes self-management education (DSMES) for adults with type 2 diabetes at four to five important times; at the time of diagnosis, once a year, with new complications, failure to reach treatment goals, and when transitions in life and care occur [41].

## Conclusions

Hypertension guidelines have metamorphosed over the last 22 years to include a more extensive at-risk population with the adoption of various blood pressure cutoffs per population. The evolution of anti-hypertension medications has also seen huge success following multiple clinical trials. We envisage that the future of the hypertension guideline will encompass BP control targets, specifically according to ethnicity, body muscle mass, sex, and geographical location, to further individualize its clinical application in the near future.

## References

[1] Siu AL (2015). Screening for high blood pressure in adults: U.S. Preventive Services Task Force recommendation statement. Ann Intern Med.

[2] James PA, Oparil S, Carter BL (2014). 2014 evidence-based guideline for the management of high blood pressure in adults: report from the panel members appointed to the Eighth Joint National Committee (JNC 8). JAMA.

[3] Krist AH, Davidson KW, Mangione CM (2021). Screening for lung cancer: US preventive services task force recommendation statement. JAMA.

[4] Alexander MR, M M (2022). Alexander MR. https://emedicine.medscape.com/article/241381-treatment#d1.

[5] Whelton PK, Carey RM, Aronow WS (2018). 2017 ACC/AHA/AAPA/ABC/ACPM/AGS/APhA/ASH/ASPC/NMA/PCNA guideline for the prevention, detection, evaluation, and management of high blood pressure in adults: executive summary: a report of the American College of Cardiology/American Heart Association Task Force on clinical practice guidelines. Hypertension.

[6] Flack JM, Calhoun D, Schiffrin EL (2018). The New ACC/AHA hypertension guidelines for the prevention, detection, evaluation, and management of high blood pressure in adults. Am J Hypertens.

[7] Greenland P, Peterson E (2017). The new 2017 ACC/AHA guidelines "up the pressure" on diagnosis and treatment of hypertension. JAMA.

[8] Michael R Goetsch, Ethan Tumarkin, Roger S Blumenthal (2022). New guidance on blood pressure management in low-risk adults with stage 1 hypertension. J Am Coll Cardiol.

[9] Bundy JD, Mills KT, He J (2019). Comparison of the 2017 ACC/AHA hypertension guideline with earlier guidelines on estimated reductions in cardiovascular disease. Curr Hypertens Rep.

[10] Wright JT Jr, Williamson JD, Whelton PK (2015). A randomized trial of intensive versus standard blood-pressure control. N Engl J Med.

[11] Yancy CW, Jessup M, Bozkurt B (2013). 2013 ACCF/AHA guideline for the management of heart failure: a report of the American College of Cardiology Foundation/American Heart Association Task Force on practice guidelines. Circulation.

[12] Shen JI, Nicholas SB, Williams S, Norris KC (2019). Evidence for and against ACC/AHA 2017 guideline for target systolic blood pressure of < 130 mmHg in persons with type 2 diabetes. Curr Cardiol Rep.

[13] Rosendorff C, Lackland DT, Allison M (2015). Treatment of hypertension in patients with coronary artery disease: a scientific statement from the American Heart Association, American College of Cardiology, and American Society of Hypertension. Circulation.

[14] Ghazi L, Oparil S (2018). Impact of the SPRINT trial on hypertension management. Annu Rev Med.

[15] (1993). 1993 guidelines for the management of mild hypertension: memorandum from a WHO/ISH meeting. Bull World Health Organ.

[16] (1999). 1999 World Health Organization-International Society of Hypertension guidelines for the management of hypertension. Guidelines subcommittee. J Hypertens.

[17] Pyörälä K, De Backer G, Graham I (1994). Prevention of coronary heart disease in clinical practice. Recommendations of the Task Force of the European Society of Cardiology, European Atherosclerosis Society, and European Society of Hypertension.. EurHeart J.

[18] Wood D, De Backer G, Faergeman O, Graham I, Mancia G, Pyörälä K (1998). Prevention of coronary heart disease in clinical practice. Summary of recommendations of the second Joint Task Force of European and other societies on coronary prevention. J Hypertens.

[19] Collins R, Peto R, MacMahon S (1990). Blood pressure, stroke, and coronary heart disease. Part 2, Short-term reductions in blood pressure: an overview of randomized drug trials in their epidemiological context. Lancet.

[20] Lewington S, Clarke R, Qizilbash N, Peto R, Collins R (2002). Age-specific relevance of usual blood pressure to vascular mortality: a meta-analysis of individual data for one million adults in 61 prospective studies. Lancet.

[21] (2003). 2003 European Society of Hypertension-European Society of Cardiology guidelines for the management of arterial hypertension. J Hypertens.

[22] Anderson KM, Wilson PW, Odell PM, Kannel WB (1991). An updated coronary risk profile. A statement for health professionals. Circulation.

[23] Mancia G, De Backer G, Dominiczak A (2007). 2007 ESH-ESC practice guidelines for the management of arterial hypertension: ESH-ESC task force on the management of arterial hypertension. J Hypertens.

[24] Zanchetti A, Grassi G, Mancia G (2009). When should antihypertensive drug treatment be initiated and to what levels should systolic blood pressure be lowered? A critical reappraisal. J Hypertens.

[25] Liu L, Zhang Y, Liu G, Li W, Zhang X, Zanchetti A (2005). The felodipine event reduction (FEVER) study: a randomized long-term placebo-controlled trial in Chinese hypertensive patients. J Hypertens.

[26] Zhang Y, Zhang X, Liu L, Zanchetti A (2011). Is a systolic blood pressure target <140 mmHg indicated in all hypertensives? Subgroup analyses of findings from the randomized FEVER trial. Eur Heart J.

[27] Williams B, Mancia G, Spiering W (2018). 2018 ESC/ESH guidelines for the management of arterial hypertension. Eur Heart J.

[28] Hypertension: Screening, 1996 .” Archived: Hypertension: Screening (2022). Hypertension: Screening, 1996. https://www.uspreventiveservicestaskforce.org/uspstf/recommendation/hypertension-screening-1996#full%20recommendation%20start..

[29] Sheridan S, Pignone M, Donahue K (2003). Screening for high blood pressure: a review of the evidence for the U.S. Preventive Services Task Force. Am J Prev Med.

[30] (2022). Evidence for the Reaffirmation of the U.S. Preventive Services Task Force Recommendation on Screening for High Blood Pressure. National Library of Medicine.

[31] High Blood Pressure in Adults: Screening (2015).” Archived (2022). High Blood Pressure in Adults: Screening. Services Taskforce, 12 Oct.

[32] Wright JT Jr, Fine LJ, Lackland DT, Ogedegbe G, Dennison Himmelfarb CR (2014). Evidence supporting a systolic blood pressure goal of less than 150 mm Hg in patients aged 60 years or older: the minority view. Ann Intern Med.

[33] Guirguis-Blake JM, Evans CV, Webber EM, Coppola EL, Perdue LA, Weyrich MS (2021). Screening for hypertension in adults: updated evidence report and systematic review for the US Preventive Services Task Force. JAMA.

[34] Goolsby MJ (2002). National Kidney Foundation Guidelines for chronic kidney disease: evaluation, classification, and stratification. J Am Acad Nurse Pract.

[35] (2004). K/DOQI clinical practice guidelines on hypertension and antihypertensive agents in chronic kidney disease. Am J Kidney Dis.

[36] (2005). K/DOQI clinical practice guidelines for cardiovascular disease in dialysis patients. Am J Kidney Dis.

[37] (2007). KDOQI clinical practice guidelines and clinical practice recommendations for diabetes and chronic kidney disease. Am J Kidney Dis.

[38] Inker LA, Astor BC, Fox CH (2014). KDOQI US commentary on the 2012 KDIGO clinical practice guideline for the evaluation and management of CKD. Am J Kidney Dis.

[39] (2021). 10. Cardiovascular disease and risk management: standards of medical care in diabetes-2021. Diabetes Care.

[40] (2021). 11. Microvascular complications and foot care: standards of medical care in diabetes-2021. Diabetes Care.

[41] (2021). 9. Pharmacologic approaches to glycemic treatment: standards of medical care in diabetes-2021. Diabetes Care.

